# Supplementary material for: Assessment of HPV Knowledge and Awareness among Students and Staff at IBB University, Niger State, Nigeria: Implications for Health Education and Prevention
Source: Healthcare (Basel). 2024 Mar 15;12(6):665. doi: 10.3390/healthcare12060665 (PMC10970435; doi:10.3390/healthcare12060665)
Supplement: Supplementary file 1 [file healthcare-12-00665-s001.zip › healthcare-2841273-supplementary.pdf]

Supplementary File: Study Survey/Questionnaire

Variables	Number of items	Sample items	Response choices	Instructions
**For Males and Females**				
**Eligibility and consent form**	2[a]	• Is this your first time taking a survey or attending a seminar about HPV and Cervical cancer?<br>• I agree to participate in this study.	Categories for both question includes: Yes, first time taking a survey or seminar about HPV and cervical cancer; No, not the first time taking a survey or seminar about HPV and cervical cancer, and Yes; No respectively.	Select only one.
**Socio-Demographics**	10[b]<br>$Q_1 – Q_{8, 8a}$	• What is your Gender?<br>• What is your age as at last birthday? **Open Ended Qualitative Items**<br>• What is your highest level of education?<br>• Are You a Student or Staff of Ibrahim Badamasi Babangida (IBB) University Lapai?<br>• How would you rate your monthly income in dollars (USD)? Note that $100 is equal to #44,550 as at December 2022.<br>• What is your Employment Status?<br>• What is your marital status?<br>• Which country are you from?<br>• How would you describe your Ethnicity?	Categories derived from those commonly used by Statistics Nigeria and Hungary. Prefer not to answer and Not Applicable were options for some items, Age was a numeric entry.	Enter Value or Select only one.
**Sexual Intercourse Activities Questions**				
**Sexual Intercourse Activities Conditional Question**	1[c]<br>Q_9	• Have you ever had sexual intercourse?	Yes, No	Select only one.
**Sexual Intercourse Activities - Sub Section**				
**Sexual Intercourse Activities - Sub Section** [1], [2]	4[c]<br>$Q_{9a} – Q_{9d}$	• At what age did you have your first sexual intercourse experience? **Open Ended Qualitative Items**<br>• How many sexual partners have you had in your lifetime? **Open Ended Qualitative Items**<br>• Do you engage in protected/unprotected sex with your partner(s)?<br>• What kind of sex do you mostly engage in?	**For $9_a – 9_b$**: Enter value (numeric responses), Categories **for $9_c – 9_d$** includes: I engage in protected sex; I engage in unprotected sex, Vaginal sex only; Anal, Oral, and Vaginal sex respectively	Enter value or Select only one.
**Smoking and STIs are Risk Factors of HPV Infection Awareness Questions**				
**Passive/Regular Smoking and STIs** [3]	5[d]<br>$Q_{10} – Q_{14}$	• Have you ever been told by a healthcare professional or Doctor that you have been infected by any of the following kinds of STDs (Sexually Transmitted Diseases) or STIs (Sexually Transmitted Infections)?<br>• Have you ever smoked?<br>• I have/had someone who smokes cigarette in my house/workplace and I usually inhale the smoke.<br>• I am a regular smoker (I daily smoke cigarettes).<br>• I am a passive smoker (I daily inhale smoke from cigarettes when someone around me is smoking).	Categories **for 10** includes: Herpes Simplex Virus (HSV), Chlamydia trachomatis, Trichomoniasis (Also known as Trich), Gonorrhea (Also known as the clap), Syphilis, Hepatitis B, None of the above; **for 11 – 14:** Yes, No.	Select multiple or only one.
**Smokers**				
**Smokers Conditional Question**	1[e]<br>Q_{15}	• Do you currently smoke?	**For 15:** Yes, No;	Select only one.
**Smokers – Sub Questions**				
**Smokers – Sub Questions**	2[e]<br>Q_{15a}–, Q_{15b}	• How many sticks of cigarettes do you smoke per day?<br>• For how long have you been smoking?	**For 15a:** 1 – 4, 5 and above; **for 15b:** Less than a year, 1 - 5 years, 6 years and above.	Select only one.
**HPV Infection Awareness Questions**				
**HPV Conditional Question**	1[f]<br>Q_{16}	• Do you have any knowledge or heard about what HPV infection is?	Yes, No	Select only one.

**HPV Infection Awareness Questions** [4]	3[f] $Q_{16a} – Q_{16c}$	• Where have you heard about HPV Infection? **Information Source**<br>• How can HPV infection be contracted from an infected person?<br>• What are the risk factors of HPV infection?	**For 16a** categories include: Public health brochures, pamphlets, flyers, or posters, etc, Social media through Facebook or Instagram or Twitter, etc, TV or radio, Newspapers or magazines, Commercials or advertisements from pharmaceutical companies, Hospital through Doctors or nurses, or other health care provider(s), Family member(s) or Friend(s) or peer or colleague, Self-search on the internet**; for 16b:** Anal sex, Vaginal sex, Oral sex, Sharing of sex toys, Sharing utensils, Skin-to-mucosa contact, Sharing toilets, Skin-to-skin touch; **for 16c:** Multiple sexual partners, Blood transfusions, Cigarettes smoking, Drinking excess alcohol, Unprotected and unsafe sexual practices, Compromised immune system, Sexually transmitted infections (STI), Vaginal, anal, or oral sex practices, Poor diet (inadequate consumption of fruits and vegetables).	Select multiple or only one.
**HPV Infection Knowledge Questions (Scale)** [4]	20[f] ($Q_{16d} –$ Q_{16w})	• HPV is a virus, not a bacteria.<br>• Women cannot contract HPV infection.<br>• Men cannot contract HPV infection.<br>• In most cases, HPV infection may not cause any symptoms.<br>• HPV 16 and 18 Genotypes are High-risk HPV Genotypes.<br>• HPV 6, 11, 42 and 61 Genotypes are Low-risk HPV Genotype types.<br>• Having sex at an early age increases the risk of getting HPV infection.<br>• HPV infection usually doesn't need any treatment to clear because it goes away overtime.<br>• HPV can be contracted through sexual intercourse.<br>• HPV-related genital warts are cancerous.<br>• There are several types of HPV Genotypes (e.g. 6, 11, 16, 18, 70, 72 and 81 genotypes)<br>• HPV 16 and 18 infections can lead to cervical cancer if untreated for a long time.<br>• HPV infection can be contracted from an infected person through genital skin-to-skin contact.<br>• Some HPV genotype infections can cause genital warts.<br>• HPV infection can cause HIV/AIDS infections.<br>• Having multiple sexual partners increases the risk of contracting HPV infection.<br>• HPV infection can be cured with Antibiotics.<br>• The use of condoms reduces the risk of HPV infection.<br>• A person could be HPV infected for several years and not know it.<br>• Most sexually active people will get HPV infection at some point in their lives.	Strongly Disagree, Disagree, Neutral, Agree, Strongly Agree (5-point Likert scale)	Select only one.
**Self-reported HPV Infection**				
**Self-reported HPV Infection**	2[g] $Q_{17} – Q_{18}$	• In the past/present, I have been told by a healthcare professional or Doctor that I had HPV Genital warts infection.<br>• In the past/present, I have been told by a healthcare professional or Doctor that I had HPV 16 or 18 infection.	Yes, No	Select only one.
**HPV Vaccine Awareness Questions**				
**HPV Vaccine Conditional Question**	1[h] Q_{19}	• Do you have any knowledge or heard about HPV vaccine before?	Yes, No	Select only one.
**HPV Vaccine Awareness Questions**	2[h] $Q_{19a} – Q_{19b}$	• Where have you heard about HPV vaccine? **Information Source**<br>• Have you taken HPV vaccine before?	**For 19a** categories include: Public health brochures, pamphlets, flyers, or posters, etc, Social media through Facebook or Instagram or Twitter, etc, TV or radio, Newspapers or magazines, Commercials or advertisements from pharmaceutical companies, Hospital through Doctors or nurses, or other health care provider(s), Family member(s) or Friend(s) or peer or colleague, Self-search on the internet**; For 19b:** Yes, No.	Select multiple or only one
**HPV Vaccine Knowledge Questions (Scale**) [4], [5]	9[h] $Q_{19c} – Q_{19k}$	• One of the doses of HPV vaccine offers protection against HPV genital warts.<br>• HPV vaccine offers protection against all Sexually Transmitted Infections (STI).<br>• A woman who has been injected HPV vaccine cannot develop cervical cancer.<br>• It is important that one injects the complete dose of HPV vaccines (three doses).<br>• There are currently no training programs in my school/workplace/hospital to inform and encourage me about the importance of HPV vaccination or educate me about HPV.	Strongly Disagree, Disagree, Neutral, Agree, Strongly Agree (5-point Likert scale)	Select only one

		• I am aware that HPV vaccine helps to protect against HPV infection and HPV-related cancers (e.g. cervical cancer).<br>• HPV vaccine is not recommended for everyone above 26 years. It must have been recommended by a Doctor for people over 26 years.<br>• Teenagers between the age of 9 - 12 years and adults below or equal to 26 years can inject HPV vaccines.<br>• Teen Girls who have had HPV vaccine do not need a Papanicolaou-stained (Pap) smear screening test when they are older.		
		**HPV Vaccine Affordability and Perception/Belief Questions**		
**Affordability (Scale)** [4], [5]	2[h]<br>$Q_{20, 20a}$	• If my doctor advises that I get HPV vaccine, I will inject HPV vaccine only because it is free.<br>• If my doctor advises that I get HPV vaccine, I will inject HPV vaccine because I can afford it.	Strongly Disagree, Disagree, Neutral, Agree, Strongly Agree (5-point Likert scale)	Select only one
**Perception (Scale)** [5], [6]	3[h]<br>$Q_{20b} – Q_{20d}$	• I feel that HPV vaccine is not safe.<br>• I feel HPV vaccine is important.<br>• If I inject or have injected HPV vaccine, it may cause nausea.	Strongly Disagree, Disagree, Neutral, Agree, Strongly Agree (5-point Likert scale)	Select only one
		**HPV Testing and Preventive Measures**		
**HPV Testing (Scale)** [4], [7]	5[h]<br>$Q_{21, 21a} –$ Q_{21d}	• Do you have access to free medical care in your country/region/state?<br>• If an HPV DNA test shows that a woman does not have HPV then her risk of developing cervical cancer is low.<br>• HPV testing is used to indicate if the HPV vaccine is needed.<br>• HPV DNA test can tell how long one has had HPV infection.<br>• If a woman tests positive for HPV DNA test she will definitely get cervical cancer at some point if not treated.	**For 21:** Yes, No; **21a – 21d:** Strongly Disagree, Disagree, Neutral, Agree, Strongly Agree (5-point Likert scale)	Select multiple or only one
**HPV Preventive Measures** [4]	3[h]<br>$Q_{21e} – Q_{21g}$	• Which of the measure(s) can help to prevent HPV infections?<br>• Have you done the HPV DNA test before?<br>• Are you willing to do the HPV DNA test if your Doctor recommends it?	**For 21e**: Safe sex practice, Use of oral contraceptive pills, HPV vaccination, Avoid genital contact with infected person(s), Getting tested regularly, Use of condoms, Having a single sexual partner, I don't know; **for 21f – 21g:** Yes, No.	Select multiple or only one
		**For Females Only**		
**Gender Confirmation Question**	1[i]<br>Q_{22}	• Are you a woman?	Yes, No	Select only one.
		**Cervical Cancer Awareness on OC Pills and Parity Questions**		
**Cervical Cancer Awareness on OC Pills** [8] **and Parity**[9]	4[j]<br>$Q_{22a} – Q_{22d}$	• How often do you use Oral Contraceptives (OC) pills?<br>• Have you given birth to a child/children before?<br>• At what age did you give birth to your first child? **Open Ended Qualitative Items**<br>• How many children do you have? **Open Ended Qualitative Items**	**For 22a:** Daily, Weekly, Bi-weekly, Monthly, Yearly, Never; **for 22b:** Yes, No; **for 22c – 22d:** Enter value (numeric responses).	Enter value or Select only one.
		**Cervical Cancer Awareness Questions**		
**Cervical Cancer Conditional Question**	1[k]<br>Q_{23}	• Do you have any knowledge or heard about cervical cancer before?	Yes, No	Select only one.
**Cervical Cancer Awareness Questions** [10], [11]	3[k]<br>$Q_{23a} – Q_{23c}$	• Where have you heard about cervical cancer?<br>• Who is more likely to be exposed to developing cervical cancer?<br>• What are the risk factors of cervical cancer?	**For 23a** categories include: Public health brochures, pamphlets, flyers, or posters, etc, Social media through Facebook or Instagram or Twitter, etc, TV or radio, Newspapers or magazines, Commercials or advertisements from pharmaceutical companies, Hospital through Doctors or nurses, or other health care provider(s), Family member(s) or Friend(s) or peer or colleague, Self-search on the internet**; for 23b:** HIV-positive women, Women who don't practice safe sex (use of condoms or the likes), Women on prolonged usage of oral contraceptive pills, Genital warts infected women, Women with multiple sexual partners, HPV 16 and 18 infected women, Women who have had more than 3 - 4 full term pregnancies, Women who started having sex below 16 years; **for 23c:** Multiple sexual partners, Blood transfusions, Cigarettes smoking, Drinking excess alcohol, Unprotected and unsafe sexual practices, Compromised immune system, Sexually transmitted	Select multiple or only one

			infections (STI), HPV 16 and 18 Genotypes, HPV 6, 11, and 61 Genotypes, Socio-cultural factors (Child marriage and poverty), Family history.	
**Cervical Cancer Knowledge Questions**				
**Cervical Cancer Knowledge Questions (Scale)** [4], [11]	11[k] $Q_{24, 24a}$ – Q_{24j}	• Cervical cancer is the leading cause of cancer deaths in women, globally.<br>• HPV infection increases the risk of cervical cancer.<br>• Smoking increases the risk of cervical cancer<br>• Men can also develop cervical cancer.<br>• Cervical cancer is hereditary.<br>• HPV 16 and 18 are the HPV genotypes causing 70% of cervical cancers and precancerous cervical lesions in women.<br>• It takes 5 - 10 years for HPV 16 and 18 to develop into cervical cancer in women with weakened immune systems.<br>• It takes 15 - 20 years for HPV 16 and 18 to develop into cervical cancer in women with normal immune systems.<br>• Cervical cancer develops around the cervix uterine.<br>• Cervical cancer is often treatable.<br>• Frequent use of contraceptive pills increases the risk of cervical cancer.	Strongly Disagree, Disagree, Neutral, Agree, Strongly Agree (5-point Likert scale)	Select only one.
**Self-reported Cervical Cancer Disease and Family History**				
**Self-reported Cervical Cancer Disease & Family History** [12]	2[l] Q_{25} – Q_{26}	• In the past/present, I have been told by a healthcare professional or Doctor that I had cervical cancer.<br>• Do you know of anyone in your family who suffered or is suffering from cervical cancer disease?	Yes, No	Select only one.
**Cervical Cancer Screening Affordability, Preventive Measures, and Screening**				
**Affordability** [8]	2[m] $Q_{27, 27a}$	• If my doctor advises that I go for Papanicolaou-stained (Pap) smear screening test, I will go for it only because it is free.<br>• If my doctor advises that I go for Papanicolaou-stained (Pap) smear screening test, I will go for it only because I can afford to pay for it.	Yes, No	Select only one.
**Preventive Measures** [8]	1[m] Q_{27b}	• Which of these measures can help to prevent cervical cancer disease development?	**For 27b:** Reduction in childbirth (1 - 4 children only), Abstinence from sex, Use of condoms, Quit smoking, Limited number of sexual partners, Abstinence from oral contraceptive pills, HPV vaccination, I don't know.	Select multiple or only one
**Screening** [8]	5[m] Q_{27c} – Q_{27g}	• Which of the following can be used to detect cervical cancer disease?<br>• How often do you visit the gynecologist?<br>• Have you ever gone for the Papanicolaou-stained (Pap) smear screening test?<br>• Will you visit the Doctor for a pelvic examination or a Papanicolaou-stained (Pap) smear screening test or meet a Doctor for a consultation about Cervical cancer after this survey?<br>• I will not go for a pelvic examination or a Papanicolaou-stained (Pap) smear screening test or meet a Doctor for a consultation about Cervical cancer after this survey because it is too expensive.	**For 27c**: Papanicolaou-stained (Pap) smear screening test, Imaging tests (CT scan, PET scan,), Lab test (Advanced genomic testing of the tumor), Colposcopy, Pelvic examination, Chest X-ray, HPV DNA test, I don't know; **for 27d:** Monthly, Half-yearly, Yearly, Never; **for 27e – 27g:** Yes,No.	Select multiple or only one

Note: [a] The questionnaire is terminated if participant selects option "No" for second question; [b] Items were administered to capture socio-demographic factors of the participants (male and female); [c] These items serves as a conditional question to check participants who have ever had sexual intercourse. This section was terminated for participants who have never had sex, they selected "No" option. Participants who indicated to have had sexual intercourse were checked based on their active sexual intercourse life. [d] For all participants smoking and Sexually Transmitted Infections (STIs) was checked. This section terminated for participants who do not smoke. [e] The items in this section were only administered to participants who currently smoke. [f] Participants who selected "No" indicating not to have any knowledge about HPV, other items to measure knowledge will not appear. [g] Items were used to capture those who have had genital warts and HPV-16 and -18 infection in the past or present. [h] All items were displayed for only the participants who indicated to have any knowledge about HPV vaccine while those who selected "No" couldn't see the sub-questions. [i] This section had items administered to the female population only. The questionnaire was terminated for the male participants here. [j] The female participants were administered items to check their use of oral contraceptive pills and number of childbirth (for those who indicated to have had children). [k] Participants with knowledge about cervical cancer were administered items from this section while those who selected "No" took items from the next section. [l] The items were administered to check female participants who have had cervical cancer diagnosis or family history. [m] Items were administered to all female participants (with or without cervical cancer knowledge).

References

[1]. K. S. Louie *et al.*, "Early age at first sexual intercourse and early pregnancy are risk factors for cervical cancer in developing countries," *Br J Cancer*, vol. 100, no. 7, pp. 1191–1197, Apr. 2009, doi: 10.1038/sj.bjc.6604974.

[2]. Y. Huang, X. Wu, Y. Lin, W. Li, J. Liu, and B. Song, "Multiple sexual partners and vaginal microecological disorder are associated with HPV infection and cervical carcinoma development," *Oncol Lett*, vol. 20, no. 2, pp. 1915–1921, Aug. 2020, doi: 10.3892/ol.2020.11738.

[3]. E. Mzarico, M. D. Gómez-Roig, L. Guirado, N. Lorente, and E. Gonzalez-Bosquet, "Relationship between smoking, HPV infection, and risk of Cervical cancer," *Eur J Gynaecol Oncol*, vol. 36, no. 6, pp. 677–680, 2015.

[4]. J. Waller, R. Ostini, L. A. V. Marlow, K. McCaffery, and G. Zimet, "Validation of a measure of knowledge about human papillomavirus (HPV) using item response theory and classical test theory," *Prev Med*, vol. 56, no. 1, pp. 35–40, Jan. 2013, doi: 10.1016/j.ypmed.2012.10.028.

[5]. S. Yarmohammadi, M. Ghaffari, Y. Mehrabi, S. Mousavi, and A. Ramezankhani, "Designing and psychometric assessment of the scale of factors influencing HPV vaccine uptake behaviors in young adults," *Infectious Agents and Cancer*, vol. 17, no. 1, p. 48, Sep. 2022, doi: 10.1186/s13027-022-00461-z.

[6]. S. Perez, G. K. Shapiro, O. Tatar, K. Joyal-Desmarais, and Z. Rosberger, "Development and Validation of the Human Papillomavirus Attitudes and Beliefs Scale in a National Canadian Sample," *Sex Transm Dis*, vol. 43, no. 10, pp. 626–632, Oct. 2016, doi: 10.1097/OLQ.0000000000000506.

[7]. B. Haward *et al.*, "Development and validation of the cervical cancer knowledge scale and HPV testing knowledge scale in a sample of Canadian women," *Prev Med Rep*, vol. 30, p. 102017, Oct. 2022, doi: 10.1016/j.pmedr.2022.102017.

[8]. Y. M. Larebo *et al.*, "Awareness, Acceptance, and Associated Factors of Human Papillomavirus Vaccine among Parents of Daughters in Hadiya Zone, Southern Ethiopia: A Cross-Sectional Study," *Vaccines*, vol. 10, no. 12, Art. no. 12, Dec. 2022, doi: 10.3390/vaccines10121988.

[9]. T. Ararsa, N. Tadele, Y. Ayalew, and D. Gela, "Knowledge towards cervical cancer screening and associated factors among urban health extension workers at Addis Ababa, Ethiopia: facility based cross-sectional survey," *BMC Cancer*, vol. 21, no. 1, p. 224, Mar. 2021, doi: 10.1186/s12885-021-07952-z.

[10]. E. K. Drokow *et al.*, "Awareness of Cervical Cancer and Attitude Toward Human Papillomavirus and Its Vaccine Among Ghanaians," *Frontiers in Oncology*, vol. 10, 2020, Accessed: Dec. 24, 2022. [Online]. Available: https://www.frontiersin.org/articles/10.3389/fonc.2020.01651.

[11]. N. Mofolo *et al.*, "Knowledge of cervical cancer, human papillomavirus and prevention among first-year female students in residences at the University of the Free State," *Afr J Prim Health Care Fam Med*, vol. 10, no. 1, p. 1637, May 2018, doi: 10.4102/phcfm.v10i1.1637.

[12]. S. Shrestha and P. Dhakal, "Knowledge, Attitude and Practice Regarding Cervical Cancer Screening Among Women Attending a Teaching Hospital, Bharatpur, Chitwan," *J Family Reprod Health*, vol. 11, no. 1, pp. 18–23, Mar. 2017.
